# The avengers: SAMHD1 cooperates with MX2/MxB to defend against HIV-1

**DOI:** 10.1128/mbio.01675-24

**Published:** 2024-09-06

**Authors:** Zhi Wan, Gang Zhong, Huiqing Wang

**Affiliations:** 1 Department of Pediatrics, West China Second University Hospital, Sichuan University, Chengdu, China; 2 Key Laboratory of Birth Defects and Related Diseases of Women and Children, Sichuan University, Ministry of Education, Chengdu, China; 3 Department of Orthopedic Surgery, West China Hospital, Sichuan University/Trauma Center, West China Hospital, Sichuan University, Chengdu, China; Dartmouth College, Hanover, New Hampshire, USA

**Keywords:** human immunodeficiency virus, SAMHD1, MX2, dNTPase activity, cDNA synthesis

## Abstract

SAMHD1 is an intrinsic limiting factor that effectively prevents HIV-1 infection in macrophages, dendritic cells, and resting CD4+ T cells. Extensive studies have underscored the indispensable role of the dNTPase activity of SAMHD1 in its antiviral function by primarily depleting dNTPs in quiescent cells, thereby impeding HIV-1 cDNA synthesis. However, recent advancements in understanding posttranslational modifications of SAMHD1 have revealed specific modification site mutants that maintain their ability to reduce dNTP levels while impairing the inhibition of HIV-1 replication. Thus, the precise anti-HIV-1 mechanism of SAMHD1 remains enigmatic, necessitating a comprehensive understanding of the underlying mechanisms to develop novel therapeutic strategies targeting its antiviral activity. Recent findings by Guo et al. shed light on the role of SAMHD1 as an HIV-1 core sensor in suppressing HIV-1 infection after viral cDNA synthesis through its interaction with MX2 (H. Guo, W. Yang, H. Li, J. Yang, et al., mBio 15:e01363-24, 2024, https://doi.org/10.1128/mbio.01363-24).

## COMMENTARY

Restriction factors play a pivotal role in the host innate immune response to virus invasion by impeding viral replication during early infection stages in a nonspecific manner, thereby restraining rapid virus proliferation until the activation of adaptive immunity for subsequent pathogen elimination. The discovery of SAMHD1, the only known mammalian deoxynucleoside triphosphate triphosphohydrolase (dNTPase), as an intrinsic anti-HIV factor has provided us with a deeper understanding of why nondividing immune cells are more resistant to HIV-1 (1–3). The investigation into the antiviral mechanisms of SAMHD1 holds a significant value in elucidating the strategies employed by the immune system to combat exogenous pathogens, particularly HIV-1. The dNTPase activity is known to be indispensable for the ability of SAMHD1 to suppress HIV-1 infection. The antiviral functions of dNTPase-deficient SAMHD1 mutants are compromised. Additionally, exogenous supplementation of deoxyribonucleotides (dNs) to elevate intracellular dNTP levels also counteracts the antiviral effect exerted by SAMHD1 (4). Therefore, a preliminary model was established for the inhibition of HIV-1 by SAMHD1: the dNTPase SAMHD1 effectively reduces the concentration of dNTPs below the threshold required for viral reverse transcriptase in resting cells, including macrophages and dendritic cells. As a result, the synthesis of HIV-1 cDNA is inhibited, thereby blocking the entire HIV-1 life cycle. However, subsequent studies have revealed that the situation is more complex than initially thought. Accumulating evidence suggests that SAMHD1 undergoes multiple posttranslational modifications, particularly phosphorylation at residue T592 and SUMOylation at K595, which are crucial for its antiviral capacity. Single point mutations in either T592 or K595 significantly impair the antiviral capacity of the SAMHD1 protein while still maintaining effective downregulation of intracellular dNTPs (5, 6). These findings indicate that although dNTPase activity is important, it is not the sole determinant for SAMHD1 functions; other factors are also needed. Therefore, the specific mechanism, by which SAMHD1 resists HIV-1 infection, remains elusive.

In a study published in *mBio*, Guo et al. elucidated an interesting and important mechanism of SAMHD1 inhibition on HIV-1 infection after viral cDNA synthesis (7). Using a well-established assay to quantify the levels of distinct viral DNA forms during HIV-1 infection, the authors observed that SAMHD1 exhibited enhanced efficacy in inhibiting the 2-long terminal repeat (LTR) form of HIV-1 DNA (a crucial indicator of nuclear entry of HIV-1 cDNA) compared to suppressing the synthesis of HIV-1 cDNA in nondividing immortalized cell lines, suggesting an additional suppressive effect on HIV-1 DNA nuclear transport by SAMHD1. Subsequently, to identify the cofactor of SAMHD1, they performed coimmunoprecipitation and mass spectrometry experiments and identified a series of SAMHD1 binding proteins. Functional experiments further confirmed the crucial role of the MX2 protein as a key binding partner of SAMHD1 in regulating its antiviral activity. Interference with MX2 expression significantly impaired the antiviral capacity of SAMHD1. Interestingly, the interferon-induced protein MX2 has been demonstrated to be a crucial effector in interferon-mediated resistance against HIV-1 infection (8–10). MX2 can recognize the incoming HIV-1 core, leading to the arrest of HIV-1 DNA-related components at the nuclear entry stage, which is consistent with the finding that SAMHD1 inhibits HIV-1 DNA accumulation within the nucleus (8–10). Furthermore, it was observed that the anti-HIV-1 mechanisms of the SAMHD1-MX2 axis differ from their antiviral actions, as evidenced by the inability of MX2-resistant HIV-1 mutated viruses to evade the synergistic antiviral effect mediated by SAMHD1-MX2. These findings imply the presence of reciprocal cooperation between the two restriction factors, highlighting greater intricacy and diversity within the immune defense network than we currently understand.

Another unique aspect of this study is that SAMHD1 directly interacts with viral components and serves as an immune sensor for the HIV-1 core. In addition to cyclosporin A, CPSF6, and MX2, SAMHD1, another HIV-1 core binding protein, has been identified. During HIV-1 infection, SAMHD1 recognizes the incoming HIV-1 core and sequesters viral components onto MX2-formed molecular traps. This mechanistic insight into SAMHD1 updates the conventional model of SAMHD1-mediated suppression of HIV-1 infection, which was attributed to the regulation of dNTP levels, by elucidating its direct impact on HIV-1 replication. Notably, SAMHD1 can interact with HIV-1 gag proteins, which raises an intriguing question regarding its potential incorporation into HIV-1 virions, warranting further investigation in future studies.

SAMHD1 functions primarily in macrophages, dendritic cells, and other nondividing immune cells to fight against HIV-1 viruses. Unlike HIV-2 and specific SIV viruses, the HIV-1 virus does not encode a viral protein named Vpx, which recruits the cellular CRL4 (DCAF1) E3 ubiquitin ligase to induce SAMHD1 protein to undergo ubiquitination and degradation, thus eliminating the antiviral effect of the SAMHD1-MX2 axis in host cells. HIV-1 is not only able to infect macrophages and other cells to a limited extent but it also protects the virus from immune recognition and antiviral response activation. Moreover, given the pivotal role of SAMHD1 in maintaining innate immune homeostasis, the downregulation of SAMHD1 expression itself can trigger interferon response activation, which is unfavorable for virus replication. Whether the abandonment of effective resistance to SAMHD1 represents a favorable evolutionary strategy for the efficient transmission and pathogenesis of HIV-1 remains to be further explored. However, the identification of the SAMHD1-MX2 antiviral axis in this study is undoubtedly helpful for the development of anti-HIV-1 virus therapy. Future research should explore the activation and enhancement of the antiviral effects of the SAMHD1-MX2 axis in target cells effectively infected with HIV-1. In addition, this study provides a novel antiviral strategy. MX2, or MX1, can form molecular traps in cells that resemble a spider’s web. Any material, including proteins, peptides, or small molecule compounds capable of capturing key viral components and presenting them to MX protein-formed molecular traps, possesses potential antiviral capacity and can serve as a promising target for antiviral intervention therapy.

In conclusion, Guo et al. elucidated an unexplored facet of SAMHD1 in suppressing HIV-1 infection postviral cDNA synthesis and shed light on the collaborative role of SAMHD1 with MX2 in facilitating the sequestration of invading viral cores, thereby enhancing the inhibitory effect of MX2 on HIV-1 cDNA nuclear entry (7). Experimental investigations employing *in vivo* infection models and analyses of the antiviral activity exhibited by the SAMHD1-MX2 axis against HIV-1 replication and virulence are imperative for a deeper comprehension of host defense mechanisms against HIV-1 and a more rational design of antiviral strategies.
